# Novel Allelic Variants in the Canine Cyclooxgenase-2 (Cox-2) Promoter Are Associated with Renal Dysplasia in Dogs

**DOI:** 10.1371/journal.pone.0016684

**Published:** 2011-02-08

**Authors:** Mary H. Whiteley, Jerold S. Bell, Debby A. Rothman

**Affiliations:** 1 DOGenes Inc., Peterborough, Ontario, Canada; 2 Department of Clinical Sciences, Tufts Cummings School of Veterinary Medicine, North Grafton, Massachusetts, United States of America; 3 American Lhasa Apso Club, Conifer, Colorado, United States of America; National Institute on Aging (NIA), National Institutes of Health (NIH), United States of America

## Abstract

Renal dysplasia (RD) in dogs is a complex disease with a highly variable phenotype and mode of inheritance that does not follow a simple Mendelian pattern. Cox-2 (Cyclooxgenase-2) deficient mice have renal abnormalities and a pathology that has striking similarities to RD in dogs suggesting to us that mutations in the Cox-2 gene could be the cause of RD in dogs. Our data supports this hypothesis. Sequencing of the canine Cox-2 gene was done from clinically affected and normal dogs. Although no changes were detected in the Cox-2 coding region, small insertions and deletions of GC boxes just upstream of the ATG translation start site were found. These sequences are putative SP1 transcription factor binding sites that may represent important cis-acting DNA regulatory elements that govern the expression of Cox-2. A pedigree study of a family of Lhasa apsos revealed an important statistical correlation of these mutant alleles with the disease. We examined an additional 22 clinical cases from various breeds. Regardless of the breed or severity of disease, all of these had one or two copies of the Cox-2 allelic variants. We suggest that the unusual inheritance pattern of RD is due to these alleles, either by changing the pattern of expression of Cox-2 or making Cox-2 levels susceptible to influences of other genes or environmental factors that play an unknown but important role in the development of RD in dogs.

## Introduction

Renal dysplasia (RD) in dogs is a category of disease that is not well defined. There are numerous reports of renal dysplasia in purebred breeds of dogs in the scientific literature, but the spectrum of clinical symptoms is so varied that it is difficult to precisely define this disorder [1], [2]. Many breeds of dogs are affected with RD. This has been documented in veterinary textbooks [3] as well as case reports and articles in the scientific literature [4]–[19].

Initially, there was controversy as to whether individual case reports of this disease that seemed to fit the description of RD were inherited or acquired [1]. Within a breed, sporadic case reports with no family history made it difficult to determine a mode of inheritance. Additionally dogs can present with RD at different ages further adding complexity to the diagnosis [2]. RD in dogs has been referred to as juvenile renal dysplasia, however, because this disease is not limited to puppies under 2 years of age [20], we prefer to use the term renal dysplasia (RD). For severe cases in puppies under two years of age the term juvenile nephropathy has also been used.

The most comprehensive study of renal dysplasia in dogs was a ten-year study by Bovee [21], who followed a group of Shih tzu, a breed known to have RD [19]. Wedge renal biopsy data was collected from 52 matings and 143 dogs. Apart from the common finding of immature fetal glomeruli, other findings from this study included mineralization of the tubules, and diffuse interstitial fibrosis in the cortex and medulla. Biopsies with higher percentages of fetal glomeruli have more severe changes in other kidney tissues. Results of this study showed that the severity of the disease was highly variable amongst individuals. The severity of the disease was reflected in the percentage of fetal glomeruli found in the renal wedge biopsy. The mode of inheritance did not follow a simple Mendelian pattern. The proposed mode in inheritance by Bovee was autosomal dominant with incomplete penetrance.

Incomplete penetrance can be explained by variations in gene expression or partial loss of function of a protein. For example, low penetrance mutations in the RB1 gene (Retinoblastoma 1 gene) fall into three classes: mutations in regulatory sequences in the promoter, missense and in frame deletions that may result in partial inactivation of the RB protein, and splice mutations that could result in a reduction of mRNA levels [22]. Otterson et al., [23] demonstrated a link between partial loss of function mutations in RB1 with incomplete penetrance. Other factors including modifier genes and environment influences can also explain why individuals having the same mutation can show a varied phenotype [24].

The present study aims to determine a molecular basis of this disease. Linkage analysis was not possible because biopsy normal individuals could produce affected offspring. Thus we took a candidate gene approach. The Cox-2 mouse knockout phenotype had striking similarities with the morphological features of the disease in dogs [25].

Cyclooxygenase, the rate-limiting enzyme in the synthesis of prostaglandins (PGs), exists in two closely related isoforms, Cox-1 and Cox-2. Cox-1 is a constitutive form of the enzyme expressed in most tissues [26], [27], while Cox-2 [28] is an inducible form. Although Cox-2 is generally associated with biologic events such as injury, inflammation, and proliferation [29], the knockout mouse model surprisingly shows abnormal kidney development.

Characterization of two knockout Cox-2 strains of mice revealed striking similarities with renal dysplasia in dogs. In one strain the transcription and translation start sites were deleted. This strain gave the most severe kidney phenotype showing 100% penetrance in -/- mice. [30]. The kidneys were severely underdeveloped with an overabundance of immature glomeruli, few functional nephrons and undeveloped mesenchymal tissue. BUN (blood urea nitrogen) and creatinine were elevated consistent with renal dysfunction. The mice had a predisposition to secondary pyelonenephritis. In addition to the kidney abnormalities there were cardiac defects in 50% of the Cox-2 -/- adult mice. The homozygous null females were largely infertile.

The other strain disrupted the coding of sequences in exon 8 by insertion and deleted 104 bp of sequence in this exon that are critical for Cox-2 function [31]. This strain showed a more complex and variable phenotype, and no heart defects were reported. The kidneys were small and pale. In the mildest form the kidneys showed immature structures consisting of immature glomeruli and tubules, and sometimes were accompanied by a thinning cortex and reduced number of glomeruli compared to wild type. In more severe cases, homozygotes showed tubular atrophy, interstitial inflammation and fibrosis as well as papillary mineralization.

Further characterization of these two mouse knockout strains, showed that Cox-2 was critical for postnatal kidney development and function [32]. This would suggest a similar role for Cox-2 in the dog model, as in the newborn dog differentiation of the glomeruli is complete by 2 weeks of age, and nephrogenesis is completed by approximately 10 weeks of age [33], [34].

Recently, a mouse strain was genetically engineered to knockdown the expression of Cox-2 [32]. This strain was generated by the insertion of a neomycin resistance cassette (Neo cassette) into the middle of intron 10. The kidney pathology in this strain was compared to a genetically engineered strain that has an active site substitution mutation that shows a severe kidney phenotype as in the Cox-2 null strains. This knockdown strain shows a phenotype that is intermediate between the wild type and Cox-2 strains with severe renal pathology [35].

Cox-2 gene expression is highly developmentally regulated. In the developing rat kidney, Cox-2 mRNA and protein are first detected beginning at mid –gestation, and are found in the subcapsular epithelial structures. Cox-2 expression continues through the completion of nephrogenesis [36]. A similar pattern of Cox-2 expression has been reported during mouse development [37]. While the role of Cox-2 in organogenesis is not clear, this gene shows high expression during late gestation in the skin, heart, cartilage and the kidney in the rat [38].

The present study presents novel allelic variants in the Cox-2 promoter consisting of insertions or deletions of putative SP1 transcription factor binding sites. We discuss how these allelic variants could influence Cox-2 gene expression, and account for the unusual inheritance of RD in dogs.

## Results

### DNA sequence analysis of the canine Cox-2 gene

We DNA sequenced the entire Cox-2 gene from a clinically affected Miniature schnauzer (27% fetal glomeruli on biopsy) and a Standard Poodle (75% fetal glomeruli on biopsy). The coding sequences (Genbank Accession number HQ110882) of both of these are in 100% agreement with the public domain assembled canine genome on Chromosome 7: 22,632,147–22,638,970. The Poodle sample was heterozygous for allele 2, while the Miniature schnauzer sample was homozygous for allele 1 (see below).

Using primer set 1 described in the experimental procedures, we DNA sequenced the region immediately upstream of the ATG translation start of the canine Cox-2 gene in clinically affected samples from several dog breeds as well as DNA from clinically normal dogs. This analysis revealed small deletions and insertions of DNA sequence just upstream of the ATG start of translation present in breeds known to have RD. Four allelic variants were found from this analysis (compared to the wild type allele from the canine reference genome sequences). Three of them were found in the clinically affected samples. A forth variant, the 6 nucleotide deletion found in allele 1 has been identified in a single Gordon setter, a single Golden retriever sample and numerous Flat-coated retrievers. One of the Flat-coated retrievers, a carrier of allele 4 has been identified as a presumptive case of renal dysplasia based on blood biochemistry and urinalysis.

Figure S1A shows the allelic variants of the canine Cox-2 gene found in RD affected individuals. Numbering of the sequence in all alleles is given relative to the translation start. Allele 1 is a deletion of wild type DNA sequences. Alignment of allele 1 with the wild type sequence shows a deletion of 6 nucleotides, CCGCCG at position -73 of the canine Cox-2 gene and a deletion of 11 nucleotides at position -37. In addition to these two changes there is a SNP at position -42. This is a “T” in the wild type sequence and a “C” in allele 1. DNA from 12 different dogs with this allelic variant was sequenced and all 12 had the “C” SNP.

Allelic variant 2 is an insertion of 12 nucleotides starting at position -78 of the canine Cox-2 gene (CGCCTCCGCCTC). 7 dogs with allelic variant 2 were sequenced. None of these had the SNP described in allelic variant 1.

Allelic variant 3 is an insertion of 24 nucleotides at position -78 of the canine Cox-2 gene (CGCCTCCGCCTCCGCCTCCGCCGC). The first 12 nucleotides of this 24 nucleotide insertion is the insertion in allelic variant 2. Four dogs with this allele were DNA sequenced, and none contained the SNP of allelic variant 1.

Allele 4 is the 6 nucleotide deletion at position -73 found in allelic variant 1. Allele 4 contains the “C” SNP at position -42.

Figure S1B shows electrophoretic separation of the 5 different alleles.

### Pedigree analysis from a family of Lhasa apsos

RD is very common in dogs of the Lhasa apsos breed [19], so we performed a pedigree study from a family of Lhasa apsos along with biopsy data and genotype information (Figure S2). In some cases the genotype had to be inferred from the parents. Dogs with inferred genotypes are marked with either a?, or the possible genotypes are given. A total of 52 dogs are included in the pedigree. Of these 35 were biopsied. Sixteen of the biopsied animals had ≤10% fetal glomeruli. These dogs had no signs of kidney impairment. Seven dogs had >10% fetal glomeruli. Three of these dogs had greater than 35% fetal glomeruli and died from renal failure between 5 and 10 years of age. One of these died at 6 months of age from chronic renal failure, and was diagnosed with RD on necropsy. The other twelve dogs had a normal renal wedge biopsy.

A mating from two biopsy normal parents produced a biopsy positive female. The sire in this case was homozygous for mutant allele 2 and the dam was heterozygous wt/allele2. The biopsy positive puppy was heterozygous wt/allele2.

There were 35 Lhasa apsos in this family with both genotypic and biopsy data. Of these 34 carried at least one of the mutant alleles, 25 had biopsy findings consistent with renal dysplasia, and 9 had normal biopsy results. The other individual was homozygous for wild type alleles and had a normal renal wedge biopsy. Importantly, the mutant alleles are statistically correlated to the renal dysplasia phenotype (X^2^ = 15.2, p<0.001).

Of Lhasa apsos in this family that were homozygous for mutant alleles, 14 were diagnosed with renal dysplasia on biopsy, and 6 were normal. Of those that were heterozygous with one mutant allele and one wild type allele, 6 were diagnosed with renal dysplasia on biopsy, and 2 were normal. There was no statistical difference in the frequency of renal dysplasia between Lhasa apsos that were homozygous or heterozygous for mutant alleles (X^2^ = 0.07, p>0.5).

Taking all of this data into account, in this family approximately 60% of dogs that are either carriers, or homozygotes for mutant alleles 2 or 3 have some aspect of abnormal kidney pathology. Of these approximately 10% have clinical signs of kidney disease which in this example reduced the life span of three, and one died as a puppy. 40% have a normal biopsy, but can produce biopsy positive offspring. These data show a dominant mode of inheritance of these alleles with incomplete penetrance and variable phenotypic expression.

### Association of RD with clinical cases in purebred breeds

Apart from the specific examples mentioned above we genotyped clinical samples from 17 other breeds that met the criteria for inclusion in this study as listed in the methods and materials section. Table 1 shows the genotype of clinical cases of RD in various breeds. All cases contained one or two copies of a mutant allele. In some of the cases, the genotype was inferred from the parents. No dogs affected with renal dysplasia were identified with a homozygous wild type genotype. Therefore these allelic variants in the canine Cox-2 gene are associated with this disease in these breeds. Table 2 summarizes the results of these findings from the cases with an unequivocal genotype.

**Table 1:** Genotypes of RD clinical samples from various breeds.

Case #	Breed	Diagnosis	Genotype	Comments
1	Poodle	Biopsy +	Allele 2/WT	Deceased from RD-75% fetal glomeruli
2	Poodle	Biopsy +	Allele2/WT	5% Fetal glomeruli
3	Shih tzu	Biopsy +	Allele1/WT	Deceased from RD<6 months
4	Gordon setter	Biopsy +	Allele1/WT	Deceased from RD
5	Gordon setter	Biopsy +	Allele2/WT	
6	Tibetan terrier	Necropsy	Allele1/WT	Deceased from RD
7	Tibetan terrier	Ultrasound	Allele2/Allele2	
8	Miniature schnauzer	Biopsy +	Allele1/Allele1	
9	Boxer	Ultrasound	Allele3/WT	Deceased from RD
10	Bernese mountain dog	Necropsy	Allele3/WT or Allele3/3	Progeny produced [2] –one was carrier Allele 3: one homozygous Allele 3- genotype inferred from parents
11	Portuguese water dog	Ultrasound	Allele3/wt	
12	Bichon Frise	Ultrasound/Biopsy	Allele3/wt	
13	Australian shepherd	Necropsy	Allele2/Allele2	Deceased from RD
14	Shetland sheepdog	Ultrasound/blood biochemistry	Allele2/Allele2	
15	Labrador retriever	Ultrasound	Allele2/Allele3	Deceased from RD
16	Yorkhire terrier	Necropsy	Allele3/wt	Deceased from RD
17	Soft –coated wheaten terrier	Ultrasound/necropsy	Allele3/Allele3 or Allele3/Allele2	Genotype of parents: sire Allele3/Allele3Dam: Allele3/Allele2
18	Soft –coated wheaten terrier	Necropsy	Allele3/Allele3 or Allele3/wt	Dam: Allele3/Allele 3
19	Soft –coated wheaten terrier	Necropsy	Allele3/wt or Allele2/wt	Dam: Allele2/Allele3: Sire: wt/wt
20	Airedele terrier	Biopsy	Allele3/Allele3-	Deceased from RD- genotype inferred from parents
21	Goldendoodle F1 cross	Biopsy/ultrasound	Allele1/Allele3 -	Deceased from RD – genotype inferred from parents
22	Rottweiler	Ultrasound	Allele2/Allele2	

Animals whose genotype was inferred from the parents are noted in the comments.

**Table 2:** Summary of results from clinical cases with unequivocal genotype.

Genotype	#Cases
1/wt	3
2/wt	4
3/wt	3
1/1	1
2/2	4
2/3	1
2/3	1
wt/wt	0

### German shepherd dog analysis

We were unable to find any cases of RD in the German shepherd dog population in the scientific literature. However a few cases were recently identified in the population in Great Britain. We obtained the pathology reports for two littermates that were under 6 months old. The diagnosis was Juvenile nephropathy, a term which is equivalent to renal dysplasia. Of twenty random dogs tested for the mutation, one carrier of mutant allele 1 was identified. The sire of this dog was one of the animals that had produced RD affected puppies, and was inferred as having at least one copy of mutant allele 1 based on examination of the remainder of the littermates of the carrier dog that was identified. These had puppies that were carriers of allele1 and homozygotes of allele1. The rest of the German Shepherds in this initial survey tested as wt/wt. Subsequently, another 65 German Shepherds were tested in order to determine the frequency of mutant alleles in this breed.

Only mutant allele 1 was found in this population and the frequency of this allele was 8% (Table 3).

**Table 3:** Frequency of the mutant alleles in various breeds.

Breed	#tested	WT	Allele1	Allele2	Allele3	Allele4	Frequency mutant alleles
Miniature Schnauzer	184	36	329	0	3	0	90%
Lhasa apso	82	27	2	73	62	0	84%
Poodle	219	154	40	234	10	0	64%
Shih tzu	93	78	91	0	17	0	59%
Boxer	208	268	84	1	63	0	36%
Shetland sheepdog	75	100	0	46	4	0	33%
German Shepherd	85	156	14	0	0	0	8%

### A case study in English cocker spaniels

Familial Nephropathy (FN) is a juvenile-onset fatal kidney disease, which has been well documented in the English cocker spaniel breed and is caused by a conserved mutation in the canine COL4A4, gene [39]. This mutation is inherited as a recessive trait. Affected individuals show a distinctive ultrastructural lesion in the glomerular basement membranes.

There have been recent reports of RD in English Cocker spaniels [40]. We were able to obtain DNA from a formalin preserved kidney sample from an RD affected English cocker spaniel. The affected puppy tested as homozygous for mutant allele 1. Both parents of this puppy were genotyped as clear for FN.

### Breed statistics

Table 3 summarizes the frequency of the Cox-2 mutant alleles in various breeds for which there were sufficient number of dogs tested.

## Discussion

The molecular basis is renal dysplasia in dogs has been elusive because of the unusual mode of inheritance and highly variable phenotype coupled with the difficulty in obtaining samples from owners who are willing to have their dogs undergo an invasive procedure for diagnosis. In this study we chose to examine the canine Cox-2 gene for mutations based its role in kidney development in the mouse. We show that allelic variants in the canine Cox-2 promoter are associated with the disease, and postulate how these DNA sequences could influence the expression of Cox-2 thereby explaining the mode of inheritance, and variable phenotype.

Perhaps the most surprising result from the characterization of Cox-2 knockout strains of mice was the presence of dysplastic kidneys consisting of embryonic tissue types. There are two related Cox-2 genes in the mouse that participate in the synthesis of prostaglandins. The Cox-1 isoform is expressed constitutively, while Cox-2 is inducible in response to inflammation following exposure to growth factors, lymphokines, or other mediators of inflammation [41], and promotes this process. Therefore, it seemed unusual that this gene would be so critical in development. However, after the initial characterization of the Cox-2 knockout mouse strains Cox-2 was shown to be highly expressed in the kidney during late gestation in the rat, reinforcing the role of Cox-2 in kidney development [38].

The dog RD model shares morphological features with both of the Cox-2 knockout mice strains The pathology seen in the dog model shows immature glomeruli and tubules in mild cases, and in severe cases, a thinning cortex, interstitial fibrosis and inflammation as well as mineralization of the tubules. As in the mouse model in severe cases the kidneys appear small and pale.

These similarities in the phenotype of renal dysplasia between mice and dogs prompted us to look for mutations in the canine Cox-2 gene.

Here we report small insertions and deletions just upstream of the ATG translation start site of the canine Cox-2 gene associated with RD. All of the mutant alleles are inherited as dominant with incomplete penetrance (see Figure S1 and Table 1). These alleles were shown to have 100% correlation with clinical cases of RD in 19 breeds. Therefore, this research suggests a similar conserved role of Cox-2 during kidney development in the dog.

The breeds with the highest incidence of RD had the highest frequency of mutant alleles while as expected the breed with the lowest frequency of mutant alleles also has the lowest incidence of disease (Table 2).

We also present data that shows that the mode of inheritance of RD is dominant with incomplete penetrance, through extensive pedigree analysis of a family of closely related Lhasa apsos, and clinical samples from many breeds of dogs. The percentage of RD individuals in this family was approximately 10%. This is consistent with the finding of Bovee [21] who estimated that the penetrance in the Shih Tzu breed was between 5 and 10%. Before this study, Bovee was able to prove a dominant mode of inheritance by an outcross breeding of a Shih Tzu with 2% fetal glomeruli with a normal Poodle mix resulted in a litter with all being affected (4–l0% fetal glomeruli). One of our clinical cases was an F1 Goldendoodle (the dam was a Golden retriever, and the sire was a miniature poodle). This confirms the finding of the outcross from the Bovee study. A mode of inheritance that is dominant with incomplete penetrance allows for a high frequency mutant alleles as seen in this pedigree.

The focus of mutation research has recently shifted to looking for disease causing mutations in the non- coding regions that regulate gene expression [42]. This is largely due to recent advances in genomics including extensive SNP maps and genome-wide association studies (GWAS). Scientists have increasingly been looking for mutations in promoter and regulatory region of genes identified by linkage or GWAS in cases where the biology of suitable candidate genes relates to the phenotype but there are no disease causing mutations in the coding regions [43]. In the present study, the unusual mode of inheritance including low penetrance suggested that candidate gene analysis was more appropriate than linkage or GWAS. Not only does the biology of Cox-2 in kidney development support our hypothesis that these promoter allelic variants are responsible for RD in dogs, but the fact that the mutations are located just upstream of the ATG translation start site justifies the mode of inheritance strengthens our argument as mutations that are dominant with incomplete penetrance, can be mutations in regulatory sequences. From the few well studied examples of promoter mutations, altered gene expression has been confirmed, and in all these cases examples the DNA sequences that are disrupted or mutated are transcription factor binding sites [43].

Recently researchers have discovered that random fluctuations in gene expression caused by a mutation in a tightly regulated developmental pathway can also lead to incomplete penetrance by upsetting stable and buffered gene expression in a wild type background [44]. As a result of these fluctuations, certain genes in the pathway fail to reach the threshold for completion of development. Given that Cox-2 is highly developmentally regulated, it is conceivable that these allelic variants could upset the balance of gene expression in a developmental pathway as described above. If this were true, RD would be considered a monogenic trait. Accordingly, since these mutations are behaving the same in different breeds of dogs, and therefore different genetic backgrounds, this model could apply to the Cox-2 allelic variants presented in this research.

Presumably, since the mouse knockout model constitutes a null mutation for the mouse Cox-2 gene and results in a phenotype that is similar to the canine RD model, it is likely that the changes in the promoter region of the canine Cox-2 results in reduced amounts of Cox-2 protein. Therefore, the most likely consequence of these variants is down regulation of gene expression. All of the allelic variants presented in association with RD involve potential SP1 transcription factor binding sites, either an SP1 site deletion or insertion of a VNTR (variable number of tandem repeat) motif containing multiple SP1 sites. The Sp1 transcription factor is a ubiquitously expressed, zinc finger containing DNA binding protein [45].

In one allele, there is a deletion of CCGCCCGCGCT at position -37. We identified a conserved sequence, CCCGCG, within this deletion by comparing the promoter regions of the dog, cow and horse Cox-2 genes. This sequence is a potential SP1 binding site. On further examination of the canine Cox-2 gene, we were unable to identify a TATA box. Often promoters that are lacking a TATA box contain multiple SP1 sites [46], [47], and this is the case for the canine Cox-2 gene. The other deletion in allele 1 is CCGCCG at position -73 is also a putative SP1 transcription factor binding site. This second deletion at position -73 is also found in allele 4. We identified this allele in a Gordon setter, Golden retriever, and several Flat-coated retrievers. One of the Flat-coated retrievers was presumed to be a case of RD based on blood biochemistry and urinalysis. We need to obtain additional clinical samples from this breed that have been diagnosed with RD by the inclusion criteria for this study in order to determine if this allele is also associated with the disease. Since SP1 is an activator of transcription [45], the deletion of an SP1 site should result in down regulation of gene expression.

The other two alleles that are associated with RD are multimers of the sequence GCCTCC. Again, this is a putative SP1 binding site.

While it is obvious that deletion of an SP1 transcription factor binding site could result in down regulation of Cox-2 as in the case of allelic variant 1 there is also justification that the insertion of multiple SP1 sites could also result in down regulation (allelic variants 2 and 3). There are examples in human disease of associations of variable number of tandem repeats (VNTR) polymorphisms containing SP1 binding sites in the promoter region [48], [49]. These examples are analogous to allelic variants 2 and three. In one such example a VNTR in the promoter of the human X-ray repair cross-complementing 5 (XRCC5) gene is a risk factor for bladder cancer. In vtiro transcription assays of the VNTR polymorphism from the XRCC5 gene showed that higher number of repeats gave lower gene expression. These authors postulated that while SP1 is generally thought of as a transcriptional activator, it can also function as negative regulator of gene expression, and that multiple copies of SP1 binding sites in the VNTRs in the promoter could result in lower gene expression.

We performed relative RT-PCR the levels of Cox-2 from kidney samples from adults with RD (data not shown). These were from dogs with severe cases of RD (75%FG, 35%FG and 44%FG). The results were initially surprising in that Cox-2 expression in the normal adult kidney is barely detectable [50], and in these clinical samples there were very high levels of Cox-2 expression. However given that inflammation is a part of this disease process [20], it was unclear if Cox-2 expression was in response to this, or was a consequence of the allelic variants. Clearly in vitro expression data would be needed to elucidate the effects of the insertions in allelic variants 2 and 3 on fetal Cox-2 expression, but this is beyond scope of this study. Studies involving promoter mutation are scarce because the studies that define the relationship between the mutation and the disease are laborious and the effect of this class of mutation can be subtle [43].

The biopsy study presented in the family of Lhasa apsos re- emphasizes the focus of this disease. Many veterinarians and dog breeders have in the past viewed RD as a disease of young puppies and have focused their attention on these cases. This is why the term juvenile renal dysplasia has become so pervasive. It seems obvious that this would be the case as these devastating case reports are more likely to be reported to the breeder and noticed by veterinarians, as it is unusual to consider kidney failure of a dog between 5 and ten years of age to be a developmental defect. The pedigree study in Lhasa apsos highlights this issue in that three individuals in this pedigree with greater than 35% fetal glomeruli died of renal failure later in life. One individual with >40% fetal glomeruli died at 8 years of age. If this animal had not had a renal wedge biopsy as a puppy, this case may have been overlooked as part of the spectrum of this disease. Therefore, this research highlights the importance of wedge biopsy data in the true diagnosis of RD. This research however provides a tool for the breeding management of this disease in canine populations that exist today.

Given the mode of inheritance a specific DNA-based genetic test is necessary for disease management in many canine breeds. Before the development of this genetic test diagnosis of RD was by wedge biopsy, an invasive procedure. However, even with this, biopsy negative animals have been shown to produce RD affected progeny as shown in this study, and the study of Bovee [21]. Therefore successful management and elimination of this disease is only possible with a diagnostic test. These autosomal dominant alleles are statistically associated with susceptibility to develop RD. While it is not known what other genetic and/or environmental factors must also be present for the development of RD, genetic testing for these mutant alleles can assist those with breeds and families at high risk of developing clinical disease.

## Materials and Methods

### DNA sequencing of the canine Cox-2 gene from clinical cases of RD in various breeds

Primers from the canine Cox-2 gene (reference sequence:Chromosome 7: 22,632,147-22,638,970) were chosen from the upstream promoter region and sequences flanking the 10 exons. These were used to directly amplify the upstream region and coding sequences of the canine Cox-2 gene. Standard PCR conditions were used for all of the fragments except for the upstream regions, which are GC rich, and required the addition of 1 X Hi-spec buffer (Bioline). PCR fragments were directly sequenced by Eurofins| MWG| Operon of Huntsville AL.

The following primer sets were used to amplify the canine Cox-2 gene:

Set1:5′UTR-exon1-part of intron1:5′ - TTG TCA AAC AAC TTG CAG CGA GCG - 3′ (forward) 5′ - ATC ACC CAG CCG AGG AGT C – 3 (reverse).

Set 2: Exons 2 and 3 and flanks: 5′ - CCT GGT TGA ACG TTG TTG GCC TTA - 3′ (forward): 5′ - CCC ACT CAG GTT CAT TCT CTC A - 3′ (reverse).

Set 3: Exon 4 and flanks: 5′ - CCA TGG ACC ACT GGT TTA CAA TAG G - 3′ (forward): 5′ -(forward): GAG ATT CAC AGA TAT CCT CAA GCA - 3′ (reverse).

Set 4: Exon 5 and flanks: 5′ - CTC CTG TAA GTG AAG AAA GCC C - 3′ 5′ - CTC CTG TAA GTG AAG AAA GCC C - 3′

Set 5: Exon 6 and 7 and flanks: 5′ - ACT ATT TAG TGG TTG TGA GAG AAA CG - 3′ 5′ -(forward): AGT AAC ATG CCA GCT TTC TCT GGG - 3′ (reverse).

Set 6: Exon 8 and flanks: 5′ - ACA AGA TTG CAT TTC AGT TGC TTG - 3′ (forward): 5′ - CAG AAA GAT CAC TTT GGT GGC AGA - 3′ (reverse).

Set 7: Part of Exon 9 intron 9 and part of exon 10: 5′ - GCA TTA GTC TTC CCT CCT TTG TAC CC - 3′ (forward):- ACC ATG GTC TCA CCA AAG ATG GCA - 3′ (reverse).

Set 8: EXON 10 and flanks: 5′ - GTT GAA AGG GAA TTG AGC AAA GGG - 3′ (forward): 5′ - CAG GCT TCT ATA GTT CAG TTG ACC G - 3′ (reverse).

### PCR conditions for a direct DNA test for RD susceptibility

DNA was isolated from buccal swabs by the method of Richards et al., [51].

The primers used to amplify all of the alleles were:5′ - ACA GCG CCT GCC TCC TCC A - 3′ (forward): 5′ - AGG TAC CCA CCT GCG CGG ACG A - 3′ reverse. These primers surrounded all of the mutant alleles and therefore diagnosis was done in a multiplex PCR reaction. Conditions for this were as follows: one cycle at 95°C for 5 minutes followed by 5 cycles of 95°C 30 s, 54°C for 30 s, and 72°C for 30 s. A further 30 cycles were performed as follows: 95°C for 30 s, 57°C for 30 s and 72° for 30 s. A final cycle of 72° for 5 minutes completed the PCR. The cycles at the lower temperature were necessary for Allele 3 would sometimes drop out of the reaction at the higher temperature. Standard PCR conditions were used with the addition of 1 X Hi-spec additive (Bioline). This was added due to the GC content of this fragment. The optimal primer concentration was 1 uM.

### Collection of wedge biopsy data from a Lhasa apso family

All biopsies done on the Lhasa apso family were sent to the University of Pennsylvania, School of Veterinary Medicine, Laboratory of Pathology, 3800 Spruce Street, Philadelphia, PA 19104-6051 For consistency, a single pathologist, Dr. Michael Goldschmidt, read the results.

### Collection of clinical samples from various dog breeds

Owners of animals diagnosed with RD submitted buccal swab samples along with a transfer of ownership and release document for the DNA. The breeds that were included are listed in Table 1.

### Inclusion criteria for the study

Dogs included in the study either had a biopsy or necropsy diagnosis of RD, or an abnormal kidney ultrasound consistent with RD.

### Participant Requirements

Participants submitting DNA samples and clinical information were required to sign a transfer of ownership and release document for the materials submitted. The owners who provided renal wedge biopsy data did so voluntarily. All renal wedge biopsies preformed by a licensed veterinarian practitioner of their choice.

## Supporting Information

Figure S1 **Allelic variants in the Cox-2 promoter.** (A) Allelic variants in the Cox-2 promoter, just upstream of the ATG translation start site. Allelic variant 1 has DNA sequences that are deleted and are boxed with the number 1 below the box. Allelic variants 2 and 3 are insertions of sequence. Allele 2 is underlined and not shaded. Allele 3 is a further insertion of sequences at nucleotide -78 relative to the ATG start, and is a duplication of the inserted sequences in allele 2. Allelic variant 4 is a deletion of 6 nucleotides CCGCCG and is boxed and marked with the number 4 below the box. (B). Gel separation of the Allelic variants of the canine Cox-2 gene. Lane 1 is the 100 bp maker (New England Biolabs); Lane 2, 3, 4, 5 are allelic variants, 1, 2, 3 and 4. Lane 6 is the wild type allele. The DNA was separated on a Spreadex-400 pre-cast gel (Elchrom scientific), using 1 X TAE buffer. The gel was run at 150 V for one hour followed by an hour at 100 V.
(TIF)

Figure S2 **Pedigree analysis of a family of Lhasa apsos.** The symbols shown in the pedigree are given at the bottom of the figure. The genotype of the dogs in the pedigree was determined either directly or by inference. Where two genotypes are possible by inference, both are listed.
(TIFF)
