# Expression of Concern: Novel Allelic Variants in the Canine Cyclooxgenase-2 (Cox-2) Promoter Are Associated with Renal Dysplasia in Dogs

**DOI:** 10.1371/journal.pone.0049703

**Published:** 2012-11-08

**Authors:** The PLOS ONE Editors

After the publication of this article, a number of concerns were raised in relation to different aspects of the research reported. The *PLOS ONE* editors carried out an evaluation of the history of the manuscript, which revealed that due to a failure in the peer review process, several aspects of the research were not adequately evaluated before publication.

As a result, the *PLOS ONE* editors have undertaken a thorough re-examination of this study, involving both external and internal advisers. This assessment has revealed the following concerns regarding the study:

- The description of the alleles in the article is inadequate.
- There are concerns over the study design employed to study the association; a single-gene association study based on Cox-2 or a genome-wide association study have been recommended as more appropriate approaches to study this association.
- The validity of the control population employed in the study is compromised as it involved a different breed.
- There are concerns about the strength of the evidence shown to support an association between the Cox-2 variant and the dogs' phenotypes, as the evidence from other breeds suggests that this may be a neutral DNA variant.

In the light of the concerns outlined above, the *PLOS ONE* editors are issuing this Expression of Concern in order to make readers aware of the concerns about the reliability of the results and conclusions reported in the article.

