# Supplementary figures and images for: Novel Allelic Variants in the Canine Cyclooxgenase-2 (Cox-2) Promoter Are Associated with Renal Dysplasia in Dogs
Source: PLoS One. 2011 Feb 8;6(2):e16684. doi: 10.1371/journal.pone.0016684 (PMC3035645; doi:10.1371/journal.pone.0016684)

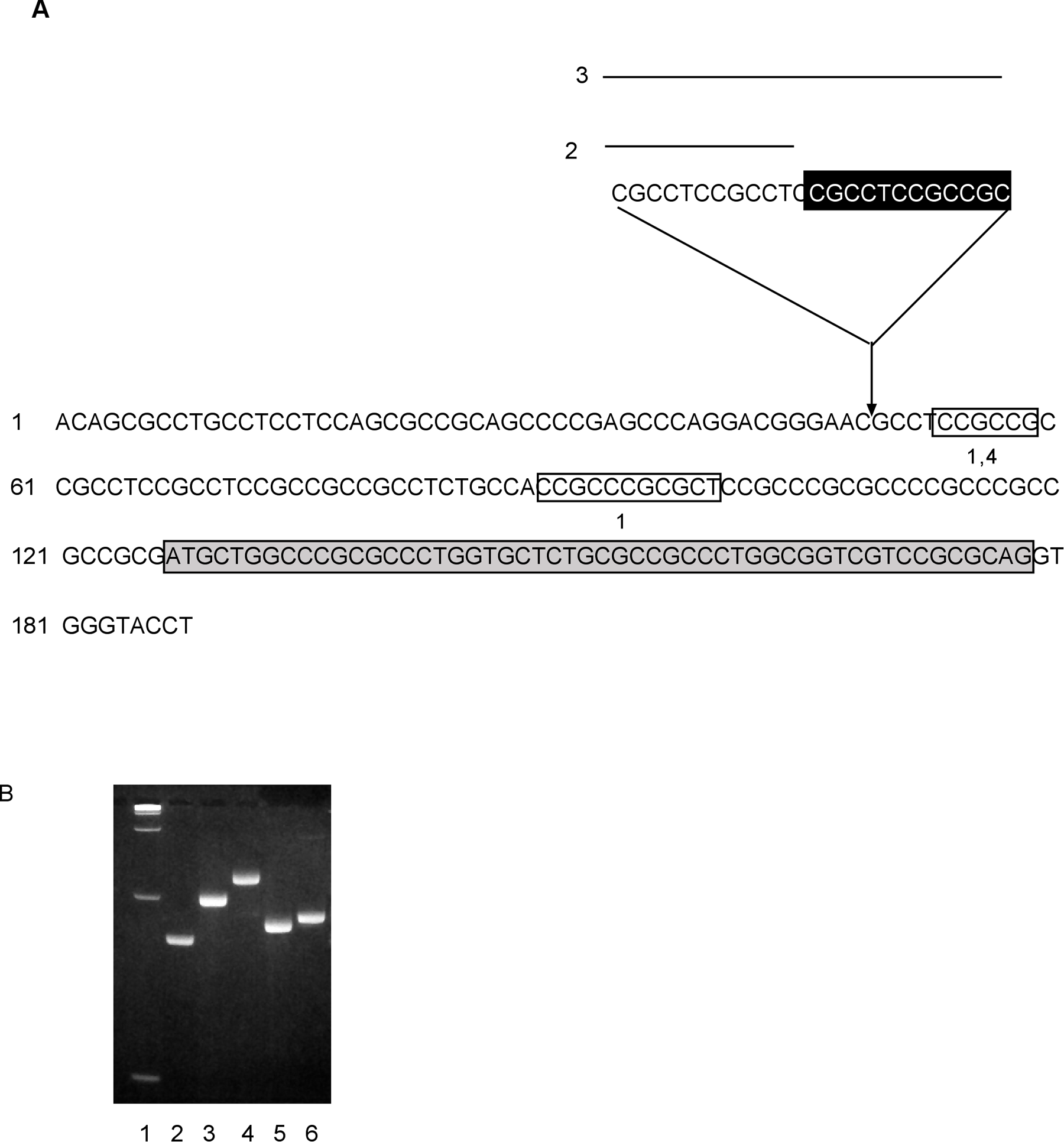

Supplement: Figure S1 — Allelic variants in the Cox-2 promoter. (A) Allelic variants in the Cox-2 promoter, just upstream of the ATG translation start site. Allelic variant 1 has DNA sequences that are deleted and are boxed with the number 1 below the box. Allelic variants 2 and 3 are insertions of sequence. Allele 2 is underlined and not shaded. Allele 3 is a further insertion of sequences at nucleotide -78 relative to the ATG start, and is a duplication of the inserted sequences in allele 2. Allelic variant 4 is a deletion of 6 nucleotides CCGCCG and is boxed and marked with the number 4 below the box. (B). Gel separation of the Allelic variants of the canine Cox-2 gene. Lane 1 is the 100 bp maker (New England Biolabs); Lane 2, 3, 4, 5 are allelic variants, 1, 2, 3 and 4. Lane 6 is the wild type allele. The DNA was separated on a Spreadex-400 pre-cast gel (Elchrom scientific), using 1 X TAE buffer. The gel was run at 150 V for one hour followed by an hour at 100 V. (TIF) [file pone.0016684.s001.tif]

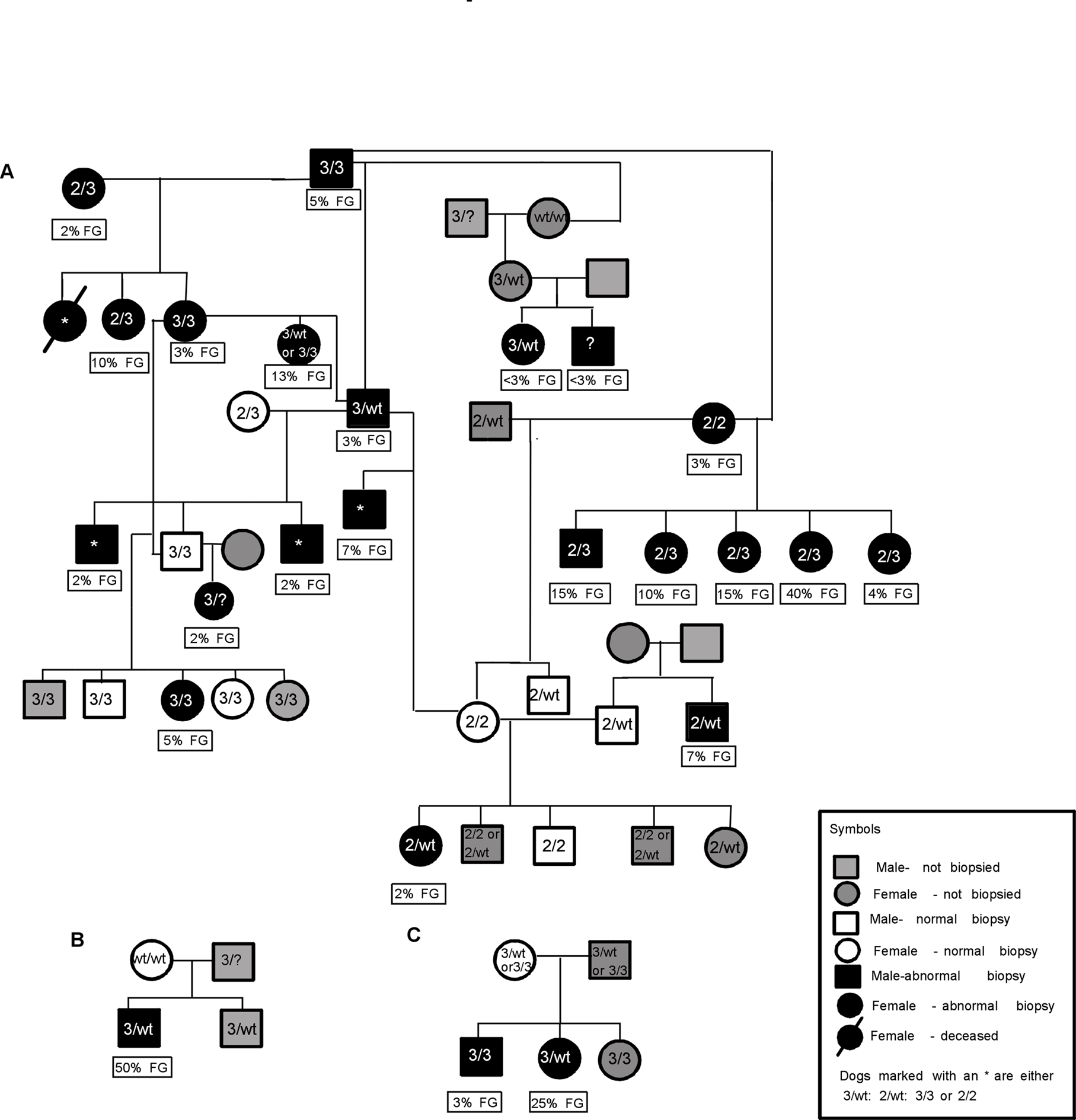

Supplement: Figure S2 — Pedigree analysis of a family of Lhasa apsos. The symbols shown in the pedigree are given at the bottom of the figure. The genotype of the dogs in the pedigree was determined either directly or by inference. Where two genotypes are possible by inference, both are listed. (TIFF) [file pone.0016684.s002.tiff]
